# To Identify Predictors of Central Lymph Node Metastasis in Patients with Clinically Node-Negative Conventional Papillary Thyroid Carcinoma

**DOI:** 10.1155/2016/6109218

**Published:** 2016-12-15

**Authors:** Jiru Yuan, Gang Zhao, Jialin Du, Xiaoyi Chen, Xiaodong Lin, Zhengbo Chen, Zeyu Wu

**Affiliations:** Department of General Surgery, Guangdong General Hospital, Guangdong Academy of Medical Sciences, 106 Zhong Shan Second Road, Guangzhou, Guangdong Province 510080, China

## Abstract

*Objective*. The aim of this study was to identify the risk factors associated with central lymph node metastasis (CLNM) in patients with clinically node-negative conventional papillary thyroid carcinoma (cN_0_ CPTC).* Methods*. A total of 190 cN_0_ CPTC patients who underwent thyroidectomy with prophylactic central neck dissection (pCND) in the Department of General Surgery at Guangdong General Hospital between March 2014 and December 2015 were assessed retrospectively. The relations of CLNM with clinicopathologic characteristics of cN_0_ CPTC were analyzed by univariate and multivariate logistic regression.* Results*. The incidence of CLNM in patients with cN_0_ CPTC was 63.2% (120 of 190 cases). Univariate analysis showed that age <45 years (*P* = 0.000), tumor size >2 cm (*P* = 0.009), multifocality (*P* = 0.001), and bilaterality (*P* = 0.000) were significantly associated with the increased incidence of CLNM in cN_0_ CPTC. No significant correlations were found between CLNM and other variables such as gender (*P* = 0.150), capsular invasion (*P* = 0.973), extrathyroidal invasion (*P* = 0.616), and lymphadenectomy (*P* = 0.062). Multivariate logistic regression analysis revealed that age <45 years (*P* = 0.000), tumor size >2 cm (*P* = 0.025), and bilaterality (*P* = 0.000) were independent risk factors of CLNM in patients with cN_0_ CPTC.* Conclusions*. Metastatic disease to central compartment lymph nodes is prevalent in patients with cN_0_ CPTC. Age <45 years, tumor size >2 cm, and bilaterality are independent risk factors of CLNM, which allow for selective CND in patients with cN_0_ CPTC.

## 1. Introduction

The incidence of thyroid cancer has increased rapidly over the previous two decades, and it is surmised to become the third most common cancer in women of all ages by 2019 [1]. Papillary thyroid carcinoma (PTC) is the most common histological subtype of thyroid cancer, accounting for 80% of all thyroid malignancies [2]. According to the World Health Organization classification system, PTC with a maximum diameter of 10 mm or less is defined as papillary thyroid microcarcinoma (PTMC). PTC with a maximum diameter more than 10 mm is defined as conventional PTC (CPTC) [3]. It has been reported that PTC has an excellent prognosis with a 10-year survival exceeding 91% and 15-year survival exceeding 88% [4, 5]. However, being well known as a lymphotropic type of cancer, PTC has high tendency to metastasize to regional lymph nodes [6]. The central compartment is the most common site of lymph node metastases. In a recent prospective randomized controlled study, central lymph node metastasis (CLNM) was found during prophylactic central neck dissection (pCND) in 50% of PTC patients [7]. There is general consensus that therapeutic central neck dissection should be performed in the presence of clinical lymph node metastases in central compartment. However, both the National Comprehensive Cancer Network (NCCN) and the American Thyroid Association (ATA) do not provide clear guidelines for or against the performance of pCND in patients with PTC [8, 9]. Consequently, there still exists substantial controversy about whether pCND should be routinely performed in patients with clinically node-negative PTC (cN_0_ PTC). For surgeons, it may be a better strategy to make an appropriate decision about the necessity of pCND according to the likelihood of the presence of CLNM based on preoperative and intraoperative risk factors. Previous studies described the risk factors of CLNM in patients with cN_0_ PTC [10–12] or cN_0_ PTMC [13, 14]. However, little has been reported about predictors of CLNM in patients with cN_0_ CPTC. Therefore, the present study focused on identifying the risk factors associated with CLNM in patients with cN_0_ CPTC.

## 2. Materials and Methods

A total of 485 cN_0_ PTC patients underwent thyroidectomy with pCND in the Department of General Surgery at Guangdong General Hospital between March 2014 and December 2015. Among them, there were 295 (60.8%) cases of cN_0_ PTMC and 190 (39.2%) cases of cN_0_ CPTC, respectively. The clinical pathological data of 190 cN_0_ CPTC patients was analyzed retrospectively in this study. Preoperative assessment included ultrasonography (US), computed tomography (CT) scan, fluoro-18-deoxyglucose positron emission tomography (PET), chest X-ray, and measurement of thyroglobulin (Tg), thyroid stimulating hormone (TSH), and anti-Tg antibody levels. US was preoperatively performed to assess the lymph node status and confirm no lymph node involvement in all these patients. Because of a variety of reasons, FNA is rarely used to preoperatively diagnose thyroid carcinoma in our hospital. In our department, CT scan was used to observe suspicious invasion of the surrounding tissues or substernal thyroid cancers. PET scan was used in patients with suspected lung or bone metastases. Patients with previous thyroid or parathyroid surgery, previous neck surgery, family history of cancer, and history of neck radiation were excluded. The following information was collected from the medical records of the patients: gender, age, tumor size, bilaterality, multifocality, lymph node metastasis, capsular invasion, extrathyroidal invasion, TNM staging, recurrence stratification (RS), and postoperative complications.

In this study, there were 129 (67.9%) women and 61 (32.1%) men. The mean age was 41.0 ± 11.6 years, ranging from 14 to 82 years. There were 67 (35.3%) patients with the age of ≥45 years and 123 (64.7%) with the age of <45 years. US showed tumor diameter >2 cm in 83 (43.7%) cases and tumor diameter ≤2 cm in 107 (56.3%) cases. All patients underwent unilateral (*n* = 5) or bilateral (*n* = 185) pCND in addition to lobectomy (*n* = 2) or total/near-total thyroidectomy (*n* = 188). At our institution, total/near-total thyroidectomy was routine surgical treatment for CPTC. These two elderly patients only underwent thyroid lobectomy mainly because of concomitant severe cardiopulmonary diseases. Moreover, the results of intraoperative frozen section in these two patients showed no evidence of multifocality, extrathyroidal invasion, and ipsilateral CLNM. The central neck dissection included the comprehensive, compartment-oriented removal of all fibroadipose tissue between the trachea and carotid sheath from the hyoid bone superiorly and the upper mediastinum and the subclavian or innominate artery inferiorly. The delphian nodes and pretracheal lymph nodes should be also removed as part of such dissection. Subsequent radioactive iodine (RAI) remnant ablation therapy after initial surgery was recommended for the presence of multifocality, extrathyroidal invasion, and CLNM. Other patients took levothyroxine for TSH suppression and received regular follow-up with a physical examination.

The pathological examinations of surgical specimens were carefully performed by 3 pathologists with over 10 years of experience at our institution. Tumors were considered multifocal if ≥2 foci were found in one or both lobes. In the case of multifocal tumor, the largest dimension was used for statistical analysis. In this study, the tumor size cutoff of 2 cm was used for statistical analysis. The pathological examination revealed 55 (28.9%) cases with bilaterality, 63 (33.2%) cases with multifocality, 120 (63.2%) cases with lymph node metastasis, 60 (31.6%) cases with capsular invasion, and 19 (10.0%) cases with extrathyroidal invasion. In 120 patients with positive CLNM, the mean number of lymph nodes with disease was 3.21 ± 2.86, ranging from 1 to 14. According to AJCC pathological staging criteria [15], 150 (78.9%) patients were in stage I, 5 (2.6%) patients in stage II, 24 (12.6%) in stage III, and 11 (5.8%) in stage IVa. According to the American Thyroid Association (ATA) RS criteria [16], there were 56 (29.5%) patients with low RS, 117 (61.6%) patients with intermediate RS, and 17 (8.9%) patients with high RS, respectively.

*Statistical Analysis*. Data collection was performed using Microsoft Excel. Statistical analysis was performed using SPSS 19.0 software. Data are presented as the means ± SD. Univariate analyses by the Pearson chi-square (*χ* ^2^) test or one-way ANOVA were performed to investigate the relationships between CLNM and clinicopathologic characteristics. Multivariate analysis was performed by binary logistic regression. *P* values <0.05 were considered statistically significant.

## 3. Results

### 3.1. The Incidence of CLNM and Postoperative Morbidity

In this study, the incidence of CLNM in patients with cN_0_ CPTC was 63.2% (120 of 190 cases). There was no permanent hypoparathyroidism or permanent recurrent laryngeal nerve (RLN) palsy. As for the level of postoperative parathyroid hormone, temporary hypoparathyroidism was found in 28 (14.7%) patients. Bilateral CND was performed in all these 28 patients. However, only 12 (6.3%) patients had the symptoms of hypocalcemia. Vocal cord palsy was found in 3 (1.6%) patients. Postoperative hemorrhage developed in 6 (3.2%) patients who received emergency operation to stop the bleeding. Among the 5 (2.6%) patients suffering from chyle leakage, 2 (1.1%) patients underwent the second operation to ligate the fistula. Two (1.1%) patients had wound infection, which was restored after conservative treatment.

### 3.2. Correlations between CLNM and Clinicopathologic Characteristics of cN_0_ CPTC

Univariate analysis showed that CLNM of patients with cN_0_ CPTC was significantly associated with age, tumor size, and bilaterality. In 123 patients with the age of <45 years, CLNM was detected in 90 (73.2%) patients, while in 67 patients with the age of ≥45 years, CLNM was detected in only 30 (44.8%) patients. The difference between these two groups was statistically significant (*P* = 0.000). CLNM was more frequent in patients with ultrasonographic tumor size >2 cm (64 of 83 cases, 77.1%), compared with patients with ultrasonographic tumor size ≤2 cm (56 of 107 cases, 52.3%) (*P* = 0.009). The incidence of CLNM in patients with bilateral tumors was 83.6% (46 of 55 cases), whereas it was only 54.8% (74 of 135 cases) in patients with unilateral tumor. The difference between these two groups had statistical significance (*P* = 0.000). In 63 patients with multifocality, CLNM was detected in 50 (79.4%) patients, while in 127 patients without multifocality, CLNM was detected in only 70 (55.1%) patients (*P* = 0.001). No significant correlations were found between CLNM and other variables such as gender (*P* = 0.150), capsular invasion (*P* = 0.973), extrathyroidal invasion (*P* = 0.616), and lymphadenectomy (*P* = 0.062) (Table 1). Multivariate logistic regression analysis revealed that age <45 years (*P* = 0.000), ultrasonographic tumor size >2 cm (*P* = 0.025), and bilaterality (*P* = 0.000) were independent predictors of CLNM in patients with cN_0_ CPTC (Table 2).

## 4. Discussion

The incidence of PTC, especially of PTMC, has increased rapidly in recent decades, due to increased awareness of thyroid nodular disease and improved diagnostic accuracy of US and fine needle aspiration biopsy [3]. It is widely accepted that metastasis to regional nodes is common in patients with PTC. Lymph node metastasis has been reported to be associated with increased recurrence and compromised survival in patients with PTC [17–20]. Total/near-total thyroidectomy is the standard surgical treatment for PTC and thyroid lobectomy is considered to be performed only for low-risk PTMC patients [9]. There is a universal consensus that therapeutic neck dissection should be performed in the presence of clinical lymph node metastases. However, there is substantial controversy regarding routine pCND in patients with cN_0_ PTC. The main reasons for the controversy around pCND include its potential higher incidence of complications and uncertainty of improved oncological outcome [20–24]. Moreover, it is unknown whether the rapidly increasing incidence of PTMC has influence on the controversy. Therefore, it is important to identify the risk factors associated with CLNM in specific PTC, which may assist surgeons in making the decision whether to perform selective pCND. In this study, we focused on identifying the risk factors associated with CLNM in patients with cN_0_ CPTC.

Karatzas et al. [3] reported that 33.3% of patients with CPTC were found to have CLNM. In this study, the incidence of CLNM in patients with cN_0_ CPTC was 63.2%. Transient hypoparathyroidism is the main complication of CND. Several studies reported that the incidences of transient hypoparathyroidism varied from 13.1% to 41.2% [20, 25, 26]. In the present study, the incidence of temporary hypoparathyroidism was 14.7%. The main cause of temporary hypoparathyroidism may be devascularization of parathyroid glands during dissection. Only 6.3% patients had the symptoms of hypocalcemia. This may be explained by the fact that preventive calcium was routinely supplemented in every patient since the day after surgery. There was no permanent hypoparathyroidism in this study. pCND also has been reported to contribute to the higher rate of temporary RLN injury. However, in a study of 1,087 patients by Giordano et al. [21], no significant differences were found between thyroidectomy alone in comparison to thyroidectomy and CND concerning transient RLN palsy. To date, no studies have shown an increased risk of permanent RLN injury in patients who underwent pCND. In this study, there was no permanent RLN palsy, and transient RLN palsy was found in 3 (1.6%) patients who all recovered within 3 months after initial surgery. Our findings support that metastatic disease to central compartment lymph nodes is prevalent in patients with cN_0_ CPTC. The results also suggest that thyroidectomy followed by pCND is a safe treatment in patients with cN_0_ CPTC.

Lymph node metastases are generally regarded as the important prognostic factor for PTC patients with ages ≥45 years. However, in a study of 47,902 patients younger than 45 years with PTC, Adam et al. [17] reported that the presence and number of lymph node metastases were associated with compromised survival. Suman et al. [27] reported that ages younger than 45 years were an independent predictor of the presence of CLNM, which allowed for selective pCND in PTC. However, similar to most previous studies, this study included both CPTC and PTMC. In this study, CLNM was detected in 73.2% (90 of 123) of patients with ages <45 years, while CLNM was detected in only 44.8% (30 of 67) of patients with ages ≥45 years. The difference between these two groups was statistically significant (*P* = 0.000). We confirmed that ages <45 years was significantly associated with increased incidence of CLNM in patients with cN_0_ CPTC.

Previous studies have shown that larger tumor size was the risk factor for the presence of CLNM in patients with PTC [28–30]. However, the size cutoffs differed in those studies. Tumor size of >0.5 or 0.6 cm has been reported to be an important predictor of CLNM in patients with cN_0_ PTMC [28, 29]. Jiang et al. [30] reported that tumor size >1.5 cm was associated with increased incidence of CLNM in patients with cN_0_ PTC. Suman et al. [27] reported that larger tumors (tumor size >2 cm) were significantly associated with CLNM. In the present study, CLNM was more frequent in patients with tumor size >2 cm (64 of 83 cases, 77.1%), compared to patients with tumor size ≤2 cm (56 of 107 cases, 52.3%) (*P* = 0.009). Multivariate logistic regression analysis revealed that tumor size >2 cm was an independent predictor of CLNM in patients with cN_0_ CPTC.

The relationship between tumor bilaterality and CLNM in patients with PTC is still controversial. Qu et al. [31] reported that bilaterality was not significantly associated with CLNM in patients with PTC. However, a recent meta-analysis including 37,355 patients with cN_0_ PTC from seven countries showed that bilaterality was an important risk factor of CLNM [11]. In this study, the incidence of CLNM in patients with bilateral tumors was 83.6%, significantly higher than 54.8% in patients with unilateral tumor. The difference between these two groups had statistical significance (*P* = 0.000). Multivariate logistic regression analysis showed bilaterality was an independent risk factor of CLNM in patients with cN_0_ CPTC. No significant correlations were found between CLNM and other variables such as gender, capsular invasion, extrathyroidal invasion, and lymphadenectomy.

The first limitation of this study was the fact that it was a retrospective study from a single center, and there might have been a selection bias. The second limitation was that we could not evaluate the effect of occult CLNM identified by pCND on oncological outcomes of patients with cN_0_ CPTC, because the follow-up time was relatively short in this study.

In conclusion, metastatic disease to central compartment lymph nodes is prevalent in patients with cN_0_ CPTC. Ages <45 years, tumor size >2 cm, and bilaterality are independent risk factors of CLNM, which allow for selective CND in patients with cN_0_ CPTC.

## Figures and Tables

**Table 1:** Univariate analysis of the correlations between CLNM and clinicopathologic characteristics of cN_0_ CPTC.

	Central lymph node metastasis		*P* value
	Negative (%)	Positive (%)	
*Gender*			0.150
Male	18 (29.5)	43 (70.5)	
Female	52 (40.3)	77 (59.7)	
*Age (Y)*			0.000
<45	33 (26.8)	90 (73.2)	
≥45	37 (55.2)	30 (44.8)	
*Ultrasonographic tumor size (cm)*			0.000
≤2.0 cm	51 (47.7)	56 (52.3)	
>2.0 cm	19 (22.9)	64 (77.1)	
*Ultrasonographic tumor size (cm)*			0.102
≤1.5 cm	24 (46.2)	28 (54.8)	
>1.5 cm	46 (33.3)	92 (66.7)	
*Bilaterality*			0.000
No	61 (45.2)	74 (54.8)	
Yes	9 (16.4)	46 (83.6)	
*Multifocality*			0.001
No	57 (44.9)	70 (55.1)	
Yes	13 (20.6)	50 (79.4)	
*Capsular invasion*			0.973
No	48 (36.9)	82 (63.1)	
Yes	22 (36.7)	38 (63.3)	
*Extrathyroidal invasion*			0.616
No	62 (36.3)	109 (63.7)	
Yes	8 (42.1)	11 (57.9)	
*Extent of lymphadenectomy*			0.062
Ipsilateral CND	4 (80.0)	1 (20.0)	
Bilateral CND	66 (35.7)	119 (64.3)	

**Table 2:** Multivariate analysis of the correlations between CLNM and clinicopathologic characteristics of cN_0_ CPTC.

	*B*	SE	Wals	df	Sig.	Exp (*B*)	95% CI	
							Lower	Upper
Age	−1.401	.362	14.997	1	.000	.246	.121	.501
Tumor size	1.320	.362	13.278	1	.000	3.743	1.840	7.613
Bilaterality	2.114	.899	5.533	1	.019	8.282	1.423	48.207
Multifocality	−.265	.821	.104	1	.747	.768	.153	3.840
Constant	−1.658	.874	3.601	1	.058	.191		
